# Digital Phenotyping in Health Using Machine Learning Approaches: Scoping Review

**DOI:** 10.2196/39618

**Published:** 2022-07-18

**Authors:** Schenelle Dayna Dlima, Santosh Shevade, Sonia Rebecca Menezes, Aakash Ganju

**Affiliations:** 1 Saathealth Mumbai India

**Keywords:** digital phenotyping, machine learning, personal device data, passive data, active data, wearable device, wearable sensor, mobile application, digital health

## Abstract

**Background:**

Digital phenotyping is the real-time collection of individual-level active and passive data from users in naturalistic and free-living settings via personal digital devices, such as mobile phones and wearable devices. Given the novelty of research in this field, there is heterogeneity in the clinical use cases, types of data collected, modes of data collection, data analysis methods, and outcomes measured.

**Objective:**

The primary aim of this scoping review was to map the published research on digital phenotyping and to outline study characteristics, data collection and analysis methods, machine learning approaches, and future implications.

**Methods:**

We utilized an a priori approach for the literature search and data extraction and charting process, guided by the PRISMA-ScR (Preferred Reporting Items for Systematic Reviews and Meta-analyses Extension for Scoping Reviews). We identified relevant studies published in 2020, 2021, and 2022 on PubMed and Google Scholar using search terms related to digital phenotyping. The titles, abstracts, and keywords were screened during the first stage of the screening process, and the second stage involved screening the full texts of the shortlisted articles. We extracted and charted the descriptive characteristics of the final studies, which were countries of origin, study design, clinical areas, active and/or passive data collected, modes of data collection, data analysis approaches, and limitations.

**Results:**

A total of 454 articles on PubMed and Google Scholar were identified through search terms associated with digital phenotyping, and 46 articles were deemed eligible for inclusion in this scoping review. Most studies evaluated wearable data and originated from North America. The most dominant study design was observational, followed by randomized trials, and most studies focused on psychiatric disorders, mental health disorders, and neurological diseases. A total of 7 studies used machine learning approaches for data analysis, with random forest, logistic regression, and support vector machines being the most common.

**Conclusions:**

Our review provides foundational as well as application-oriented approaches toward digital phenotyping in health. Future work should focus on more prospective, longitudinal studies that include larger data sets from diverse populations, address privacy and ethical concerns around data collection from consumer technologies, and build “digital phenotypes” to personalize digital health interventions and treatment plans.

## Introduction

Patient engagement is a significant challenge that health care organizations face, as consumers expect and demand a more personalized approach when they seek health care services [1]. Artificial intelligence (AI)–led smart health care services are emerging as promising tools to improve the efficiency and effectiveness of health care service delivery [2]. Among these is digital phenotyping, which is the real-time collection of individual-level active and passive data from users in naturalistic and free-living settings via personal digital devices, such as mobile phones and wearable devices [3]. Personal digital devices and platforms, such as smartphones, wearable devices, and social media, offer a wealth of information about an individual’s behavior and health status. These are valuable sources of several active and passive data points, such as phone utilization metrics, GPS information, search histories, linguistic nuances in text messages, duration of sleep, step counts, calories burned, and heart rate variability. These data points can be leveraged to gain a nuanced understanding of individual behaviors to predict disease exacerbation or relapse, design a more targeted intervention, and improve decision making in clinical settings [2,3].

Digital phenotyping is an emerging field that intersects data analysis, engineering, and clinical practice, bringing about unique challenges in reporting and reproducibility. Although the advantages of a multidisciplinary approach are evident, these multidisciplinary domains have yet to be brought together efficiently to ensure standardized reporting and easier replicability [4].

The techniques and methodologies used to collect, process, and classify active and passive data in digital phenotyping vary across the literature. AI and machine learning have already driven developments in wearable sensing and mobile health; they have helped enhance human activity recognition models, improve the accuracy of predicting human behaviors, and deliver more personalized lifestyle recommendations [5]. Research points to trust, perceived usefulness, and personalization directly influencing the frequency of use of digital health care services [2].

Given the plethora of data points that smartphones and wearable sensors and devices yield, AI and machine learning can be used to process and analyze these large data sets [6]. The purpose of passive data is to improve patient monitoring and outcomes across a variety of clinical applications [7]. In a systematic review of machine learning studies on digital phenotyping across psychosis spectrum illnesses, the machine learning approaches used included random forests, support vector machines, neural nets, k-nearest neighbors, and naive Bayes classifiers [8]. Machine learning algorithms used to analyze these multidimensional data can also be used to predict risks and probabilities and make binary decisions, such as discharge versus no discharge [9]. Other computational tools that have been used for digital phenotyping include data mining and statistical methods [10].

The immense potential of digital phenotyping in the clinical landscape is gaining increasing attention, leading to a measurable increase in related published research in the past 5 years. This trend has also been observed for health and clinical research related to analyzing active and passive data from smartphones and wearable devices. Digital phenotyping perhaps demonstrates the greatest potential for precision digital health interventions. Assigning a digital phenotype can help build predictive models around user behavior, providing insights into their engagement levels and the means to optimize the efficacy of digital health interventions. This method of segmentation offers further opportunities to enhance diagnosis, risk prediction, treatment effectiveness, and patient monitoring [11]. Given the nascency of research in the digital phenotyping field, there is heterogeneity in the clinical use cases, types of data collected, modes of data collection, data analysis methods, and outcomes measured.

Thus, the primary aim of this scoping review was to map the published research on digital phenotyping and to outline study characteristics, methods of active and passive data collection, data analysis approaches used (specifically machine learning techniques, if any), and future implications. The desired outcomes of this review are to provide a broad overview of ongoing research on digital phenotyping and identify gaps and opportunities in future research and practice, especially regarding leveraging machine learning techniques for digital phenotyping.

## Methods

### Overview

We conducted this scoping review to examine the breadth of published evidence related to digital phenotyping in health care. We utilized an a priori approach for the literature search and data extraction process to ensure the search protocol was replicable. The PRISMA-ScR (Preferred Reporting Items for Systematic Reviews and Meta-analyses Extension for Scoping Reviews) checklist guided the methodology and reporting of this scoping review (Multimedia Appendix 1) [12].

### Search Terms

As the term “digital phenotype” is relatively nascent in the research landscape, we conducted a preliminary scoping of literature on PubMed and Google Scholar to identify different search terms associated with digital phenotyping. This ensured that our literature search would capture all published research related to digital phenotyping, even if the term was not explicitly mentioned anywhere in the text. These were the search terms finally used to conduct the literature search: “digital phenotyp*” OR “active data” OR “passive data” OR “digital biomarker*” OR “digital footprint” OR “mobile data” OR “mobile phone data” OR “digital sensing” OR “digital fingerprint*” OR “smartphone data” OR “wearable*” OR “wearable device*” OR “wearable data” OR “precision data.”

### Eligibility Criteria

We included peer-reviewed original research articles in English, as our aim was to explore the gaps and opportunities in scientific research on digital phenotyping. Furthermore, in line with the breakdown of the definition of digital phenotyping by Onnela [3], studies were deemed eligible if they included the following characteristics: (1) if any types of active or passive data were collected. For this review, active data referred to data that required direct input from users in response to prompts, and passive data referred to data generated and collected without inputs from the user [13]; (2) if a wearable device or mobile phone was used to collect the active and/or passive data; (3) if the terms “digital phenotype” or “digital phenotyping” were in the title, abstract, or keywords; and (4) if the active and/or passive data were classified in some ways (ie, if any “phenotypes” were established or if the data were used to make predictions regarding diagnosis, symptom exacerbation, or relapse).

We limited the years of publication to 2020, 2021, and 2022 because from our preliminary search, we conjectured that these years witnessed a sharp increase in the number of publications related to digital health, active and passive data collection, and wearable devices. Moreover, focusing on these years would provide the most recent snapshot of digital phenotyping research, as the field is rapidly and continually evolving. Table 1 shows the uptick in digital phenotyping research published in the last 5 years. This timeline was the result of using the search terms and article type filters that were part of our eligibility criteria.

We excluded reviews, meta-analyses, opinion pieces, grey literature, letters to the editor, commentaries, study protocols, articles describing phenotyping in the context of genetics, and articles not in English. We also excluded studies that solely focused on the feasibility and acceptability of interventions using digital phenotyping.

**Table 1:** PubMed timeline of digital phenotyping research published from 2017 to 2022. The timeline indicates a sharp increase in published literature from 2019 onward.

Year	Research articles published, n
2017	129
2018	173
2019	257
2020	246
2021	232
2022	114

### Sources of Evidence

We used PubMed and Google Scholar to identify relevant literature. We chose PubMed due to its focus on clinical and health-related research and Google Scholar to surface literature that intersected multiple disciplines.

We utilized additional filters on PubMed to exclude the following articles that did not meet our study type and year of publication criteria: (1) study type: clinical study, clinical trial, comparative study, controlled clinical trial, multicenter study, observational study, randomized controlled trial (RCT); and (2) results by year: from January 1, 2020, to January 18, 2022.

In Google Scholar, we filtered the results according to the date of publication. We used the custom range of 2020-2022.

### Screening Process

After applying the search terms and filters on PubMed and Google Scholar to identify relevant articles, the citations were imported into the Rayyan.ai system (Rayyan Systems Inc), a free online tool to create and manage systematic reviews. Author SDD conducted the final search and imported the citations on January 18, 2022. Then, authors SDD and SS independently screened the titles, abstracts, and keywords using the predetermined eligibility criteria. Any discrepancies regarding which articles should be shortlisted were resolved by discussions between SDD and SS. The next step of the screening process involved screening the full texts of these shortlisted articles; all reviewers were randomly assigned articles to screen for concordance with the eligibility criteria. The reviewers had regular discussions to resolve any disagreements on studies to include in the final analysis.

### Data Extraction and Charting

After the authors screened the full-text articles for inclusion in the scoping review, a Google Sheet was created to extract descriptive characteristics of the final articles. Details recorded in the Google Sheet included study title, author(s), year of publication, country of origin, study design, clinical area, active and/or passive data collected, mode of data collection, data analysis approaches, and limitations of the study.

The reviewers independently conducted the data extraction and charting of the final articles. SDD and SS were consulted for any queries regarding the data extraction and charting process that the other reviewers had. The results of the data extraction and charting process are presented in Multimedia Appendix 2.

We did not conduct a formal critical appraisal of the final articles because the primary aim of our scoping review was to describe the breadth of evidence and map the characteristics of the literature on digital phenotyping.

### Synthesis of Results

We summarized the studies for the following characteristics: countries of origin, study designs, clinical areas, active and/or passive data collected, modes of data collection, data analysis approaches, and limitations. The World Health Organization’s region classification was used to group the countries of origin [14]. The study designs were grouped as follows: observational studies, randomized trials, post hoc analyses of observational studies, and post hoc analyses of RCTs.

In this scoping review, we mapped the types of data collected in the studies into the following categories: wearable/activity (passive data), mobile phone (passive data), clinical/biometric (passive data), and active. The passive data categories were based on the Activity-Biometrics-Communication framework by Jayakumar and colleagues [15]. Wearable/activity data included those generated by and collected from wearable devices, mobile phone data included those passively collected from a mobile app or from the mobile device itself (such as the microphone), and clinical/biometric data included passively collected biological data such as blood pressure, body temperature, heart rate, and so on. Active data included patient-reported outcome measurements on a mobile app, as well as responses to survey questions on a mobile app. We tabulated all the passive and active data points collected in the included studies.

The following categories were used to map how active and passive data were collected in the included studies: wearable device, mobile app, wearable device + mobile app, wearable device + other, and other. We tabulated the wearable devices and mobile apps used in the studies. We used the following broad categories to map the data analysis approaches: regression, statistical methods, machine learning techniques, and latent growth analysis.

## Results

### Search Results

**Figure 1:**
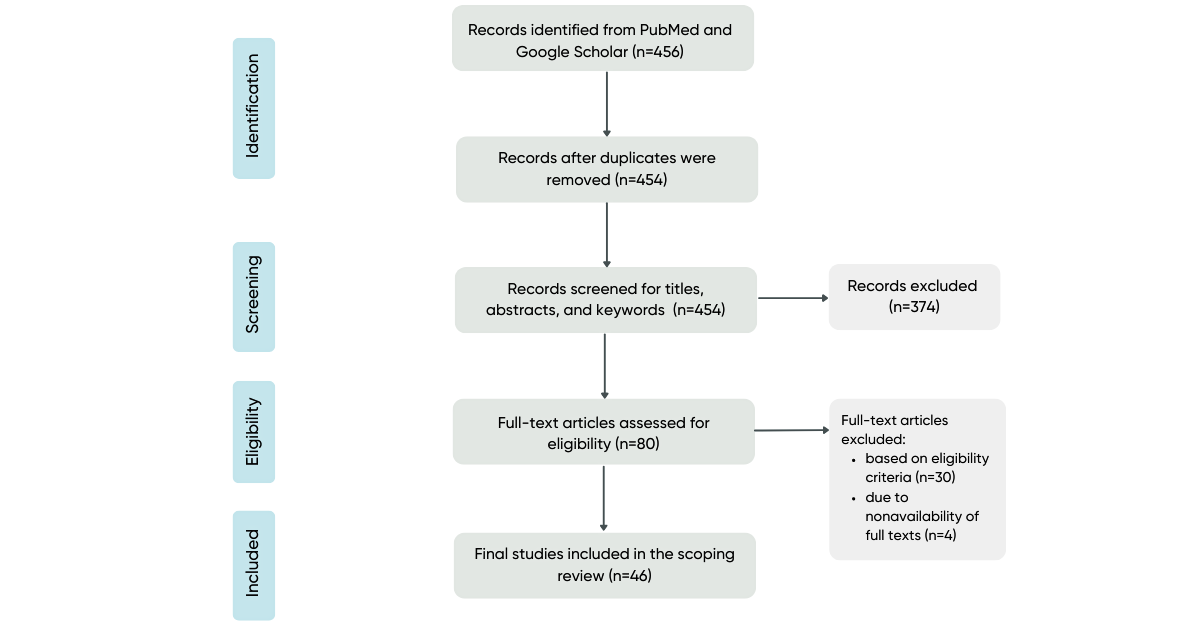
PRISMA (Preferred Reporting Items for Systematic Reviews and Meta-analyses) flowchart of the process of study identification, screening for eligibility, and final inclusion in this scoping review.

Figure 1 depicts the PRISMA flowchart of the study selection process. A total of 454 articles were identified from PubMed and Google Scholar after removal of duplicates. Following the screening of the titles, abstracts, and keywords, 80 articles were eligible for full-text review. After reviewing the full-text articles, we excluded 30 that did not meet our eligibility criteria and 4 whose full texts were unavailable. Thus, 46 articles were deemed eligible for inclusion in this scoping review. Detailed characteristics of these 46 articles are presented in Multimedia Appendix 2.

### Countries of Origin

Most studies (n=26, 56.5%) originated from North America, including the United States (n=24) [16-39] and Canada (n=2) [40,41]. Twelve studies (26.1%) were conducted in European countries, such as France [42,43], Germany [44,45], Italy [46,47], Luxembourg [43], Spain [48,49], Switzerland [50], the Netherlands [48,49], and the United Kingdom [47-49,51-53]. Six studies (13%) originated from countries in the Western Pacific region, including Australia [54,55], Japan [56,57], and South Korea [58,59]. Only 1 study (2.2%) came from the Southeast Asian (China) [60] and Eastern Mediterranean (Qatar) [61] regions. Table 2 summarizes the studies’ regions of origin.

**Table 2:** Summary of the number of studies by the World Health Organization’s region classification.

World Health Organization’s region classification	Countries of origin	Studies, n (%)
Eastern Mediterranean	Qatar	1 (2.2)
Europe	France, Germany, Italy, Luxembourg, Spain, Switzerland, the Netherlands, and the United Kingdom	12 (26.1)
Southeast Asia	China	1 (2.2)
North America	Canada, the United States	26 (56.5)
Western Pacific	Australia, Japan, South Korea	6 (13)

### Study Designs

The most dominant study design was observational (n=28, 60.9%) [17, 20, 21, 23-25, 27, 28, 31, 32, 34, 36-40, 42-47, 49-51, 57, 58, 60], followed by randomized trials (n=10, 21.7%) [19,22,26,30,35,41,52-55], post hoc analyses of RCTs (n=5, 10.9%) [18,29,56,59,61], and post hoc analyses of observational studies (n=3, 6.5%) [16,33,48].

### Clinical Areas

The clinical areas investigated in the included studies were heterogeneous. Most (n=15, 32.6%) studies focused on psychiatric disorders, mental health disorders, and neurological diseases, including Parkinson disease [44,51]. Psychiatric and mental health disorders included body dysmorphic disorder [37], disordered eating [54], cognitive impairment [61], substance use disorder [17,31], depression [40,46,48,49,53,60], anxiety disorders [40,53], schizophrenia [23], and stress [26].

A total of 7 (15.2%) studies focused on cardiovascular diseases, which included hypertension [19,21,45], hypercholesterolemia [56], heart failure [24], and general cardiovascular health [32,47]. Five studies (10.9%) focused on cancer, including skin cancer [28], melanoma [34,35], breast cancer [55], and monitoring patients undergoing chemotherapy [27]. Moreover, 3 (6.5%) focused on diabetes [30,38,52], and 7 (15.2%) focused on participants who were overweight or obese [16,18,30,33,36,43,59]. Meanwhile, 4 (8.7%) studies assessed hospital-related outcomes, including postoperative recovery [20], posthospital discharge [22,29], and in-hospital admission of geriatric patients [50]. Three studies (6.5%) included patients undergoing hemodialysis [25,46,61]. Other clinical areas investigated included circadian rhythms [42], cough [57], sarcopenia [58], physical training [39], and rheumatoid arthritis and lupus erythematosus [41].

### Types of Active and Passive Data Collected

We categorized the types of data collected in the studies as follows: wearable/activity (passive data), mobile phone (passive data), clinical/biometric (passive data), and active.

Regarding passively collected data, 37 (80.4%) studies evaluated wearable/activity data, 3 (6.5%) studies evaluated mobile phone data, and 13 (28.3%) studies evaluated clinical/biometric data. Nine (19.6%) studies assessed active data. Table 3 summarizes the wearable/activity, mobile phone, clinical/biometric, and active data points collected in the studies.

**Table 3:** List of the active and passive data points collected in the studies included in this scoping review.

Passive data			Active data
Wearable/activity	Mobile phone	Clinical/biometric	
Mobility pattern [37]	Frequency of app use [37]	Heart rate [17, 19-21, 32, 39, 43, 45, 48, 53, 60]	Exercise amount [54,59]
Ultraviolet radiation exposure [28,34,35]	Quantity of app use [36]	Skin conductance [17]	Body satisfaction [54]
Step count [18-22, 26, 27, 29, 30, 39, 43, 46, 56, 59-61]	Number of days activity monitor data were uploaded to the web-based app [52]	Skin temperature [17]	Fitness/health motives for exercise [54]
Gait parameters [44,51,58]	Call logs [60]	Blood pressure [19,21,43]	Engagement in binge eating [54]
Anticipatory postural adjustments [51]	Text message logs [60]	Movements in epigastric region [57]	Engagement in dietary restraint [54]
Sit-to-stand duration [51]	App usage logs [60]	Expansion of throat skin [57]	Immediate mood [60]
Energy expenditure [39,52]	GPS location [40,60]	Weight [38,43]	Patient Health Questionnaire-9 in an app [60]
Sleep duration [19, 26, 39, 48, 49, 53, 56, 60]	Screen on-and-off status [40,60]	Blood glucose levels [38]	Liebowitz Social Anxiety Scale [40]
Sleep efficiency [19,48,49,53,56]	Ambient audio [40]	N/A^a^	Generalized Anxiety Disorder 7-Item Scale [40]
Sleep stage [56]	Light sensor data [40]	N/A	Patient Health Questionnaire 8-item scale [40,48,49]
Distance walked [45,56]	Telephone call recipient [42]	N/A	Sheehan Disability Scale [40]
Daytime nap duration [24]	Moment in time of telephone call [42]	N/A	Responses to daily assessment [59]
Daytime nap frequency [24]	Telephone call duration [42]	N/A	Meals logged [59]
Repositioning events [36]	Articles read [59]	N/A	Intake of green foods logged [59]
Three-dimensional acceleration [17]	Comments posted [59]	N/A	Rosenberg Self-Esteem Scale [48]
Number of activity monitor wear days across the intervention [52]	Number of posts [59]	N/A	Weigh-ins logged [59]
Number of interactions with wearable sensor [17]	Messages sent to coaches [59]	N/A	Self-reported location [31]
Physical activity [16, 33, 38, 41, 45, 47, 48, 50, 52]	Number of likes [59]	N/A	Self-reported social context [31]
Number of postural transitions [61]	Screen time metrics [24]	N/A	Self-reported cannabis use [31]
Exercise time [59]	N/A	N/A	Mental and physical 5-point scale [39]
Step speed [19]	N/A	N/A	Self-reported sleep, hydration, and nutrition [39]
Time spent walking [16]	N/A	N/A	Confidence in instructors and graduation [39]
Durations of postural transitions [61]	N/A	N/A	Speech patterns [48]
N/A	N/A	N/A	Cognitive function [23,48]

^a^N/A: not applicable.

### Modes of Data Collection

The categories used to map how active and passive data were collected in the included studies were as follows: wearable device, mobile app, wearable device + mobile app, wearable device + other, and other. Most (n=25, 54.3%) studies fell under the wearable device category [16-20, 22, 24, 25, 32-34, 36, 38, 43, 44, 46, 47, 49-51, 55-58, 61]. Many (n=14, 30.4%) studies also collected data using a combination of wearable devices and a mobile app and thus fell under the wearable device + mobile app category [21,23,26-30,35,39,45,48,53,54,60]. Of the studies, 8.7% (n=4) fell under the mobile app category [31,37,40,59], 4.4% (n=2) under the wearable device + other category [41,52], and 2.2% (n=1) under the other category [42], which included data collection through web-based applications. Textbox 1 lists the types of wearable devices and mobile apps used in the studies.

**List of wearable devices and mobile apps used to collect active and passive data in the studies included in this scoping review.**

Wearable devices:

- Activity monitor (Actical, Philips Respironics) [24]
- activPAL (PAL Technologies Limited) [55]
- Apple Watch Series 2, 3, or 4 smartwatches [21,39,45]
- Biobeam wearable device [53]
- Body weighing scale (Withings) [43]
- BP-800 blood pressure monitor (Withings) [43]
- Cellular-enabled scale [38]
- E4 wearable sensor (Empatica) [17]
- FitBit [16,20,25,26,32,33,38,41,48,49,54,56]
- Garmin Vivofit2 activity monitor [55]
- Inertial SHIMMER sensors (Shimmer Research Limited) [44]
- Mi Band 2 (Xiaomi Corporation) [60]
- Microsoft Band 2 [27]
- Omron Evolv Wireless Blood Pressure Monitor [19,21]
- Phone-tethered glucometer [38]
- Withings pulse activity tracker [43]
- Samsung Galaxy Watch [19]
- SenseWear Mini (BodyMedia) multisensory monitor [41]
- SenseWear Armband [46]
- Shade wearable ultraviolet radiation sensor [28]
- Smartwatch (unspecified) [23]
- Ultraviolet radiation exposure sensor [28,34]
- Validated pendant sensor (PAMSysTM, BioSensics LLC) [61]
- Waist-worn activity tracker (ActiGraph wGT3X-BT) [34]
- Wearable smart belt (WELT) [58]
- Wearable triaxial accelerometer sensor [36]
- Wrist-worn ActiGraph GT3X+ [55]
- Wrist-worn ultraviolet dosimeter [35]
- Wrist-worn wearable device (Withings Activite Steel) [18,22,29,30]

Mobile apps:

- Apple Health app [21]
- Beiwe app [23]
- BreeConnect App [45]
- InstantSurvey smartphone app [54]
- iOS Biobase app [53]
- MApp [31]
- mindLAMP app [23]
- Mood Mirror app [60]
- Noom app (for food diaries) [59]
- Patient-reported outcomes app [27]
- Perspectives app on iOS [37]
- Withings HealthMate app [29]

### Data Analysis Approaches

Regarding the data analysis techniques, 22 (47.8%) studies used regression-based statistical methods [16,20,22,23,28,30,33,35,37,40,41,43,45,48-50,53,54,56,58,61], 2 (4.3%) used latent growth analysis [18,38], and 14 (30.4%) used other statistical analysis methods [21,24-26,29,31,32,34,42,44,46,47,52,55]. One (2.2%) study did not perform any statistical analyses because it was a case report [36]. Only 7 (15.2%) studies used machine learning approaches to build predictive models [17,19,39,51,57,59,60], while 1 study used logistic regression and random forest classifiers [51]. Another study tested 25 classification models from the following categories: decision trees, discriminant analysis, logistic regression, naive Bayes classifiers, support vector machines, nearest neighbor classifiers, and ensemble classifiers [17]. One study used 6 different machine learning models: support vector machines, k-nearest neighbors, decision trees, naive Bayes, random forest, and logistic regression [60]. A study conducted in Japan used a deep learning–based machine learning algorithm called variational autoencoder for feature extraction and k-means clustering algorithm for classification [57]. Another study used random forest, support vector machine, gradient boosting decision trees, long short-term memory, and autoregressive integrated moving average techniques [19]. A study from South Korea used an elastic net machine learning approach [59], and 1 from the United States used a random forest approach [39].

### Limitations of the Included Studies

The limitations put forward by the authors of the studies in this review were heterogenous. Most studies reported low generalizability of their findings due to small sample size, single-center study designs, short study durations, and narrow population segments included in the studies. Due to the observational nature of the studies, causal relationships between the passive and active data collected and outcome measures could not be confirmed. Some studies also reported device- and app-related limitations, including short battery life of smartwatches (leading to underestimation of physical activity) [21], challenges in keeping the app running 24/7 [60], no measurements of users’ interactions with mobile phone notifications [26], missing data [23,30,48,49], and drawbacks in the algorithms tested [16,32,45,57,58]. Another limitation reported was reliance on self-reported data, which included active data collected and those collected for outcome measurements.

## Discussion

### Principal Findings

Our scoping review provides an insight into the breadth of research on digital phenotyping published in the last 3 years. Most studies originated from North America, had observational study designs, and used wearable devices to collect passive and/or active data. The studies spanned various clinical indications, but psychiatric disorders, mental health disorders, and neurological diseases were the most common areas. Only 7 (15.2%) studies used machine learning–based approaches for data analysis, while the rest predominantly used statistical methods. Most studies had low sample sizes, limiting their generalizability to other populations and clinical settings.

Digital maturity and uptake of wearables vary significantly across regions; however, the onset of the COVID-19 pandemic has generally led to an increase in the use of digital health tools for remote monitoring [62]. In our scoping review, 56.5% (n=26) of the studies were conducted in North America. Market research trends from 2021 indicated that North America is currently leading the global digital health market, and this market is poised to accelerate even faster than the global average between 2021 and 2025 [63]. There is also a significant impact on the pace of transformation from the aftereffects of large-scale enterprise systems implementations. Consumers from this region reported an increase in wearable use from 9% to 33% over the last 4 years, while the number of smartwatch users grew from 42 million to 45.2 million users from 2020 to 2021 and is expected to reach 51.9 million by 2024 [64]. These trends point to greater personalization and innovation in the use of health monitoring tools and wearables in North America. In Europe, the adoption of digital health tools among patients increased from 85% in 2015 to 87% in 2017, with patients increasingly adopting technologies such as wearables and remote patient monitoring tools [65]. The increase in the uptake of digital tools in Europe is attributed to the growing geriatric population coupled with the rising preference for remote patient monitoring. Increasing government initiatives for the development of digital health in the region and growing digital infrastructure will drive market growth [66].

The types of studies in this review were primarily observational (n=28, 60.9%), most of which were cohort-based prospective observational studies. Since wearable device–related studies are relatively new, the rigor and complexity of the study protocols varied significantly, from randomized trials to simple observational studies. We found that digital phenotyping research has been primarily explored in clinical indications related to mental illnesses and psychiatric disorders, but several studies also focused on chronic conditions such as cardiovascular diseases, obesity, and cancer. This points toward growing attention on the real-time monitoring of chronic, long-term conditions, as the patient journeys of these conditions largely occur outside clinical settings.

We observed that the most common data collection tool used across the studies was commercial wearable devices, in line with other reviews conducted in this area [15,67]. Wearable devices have immense potential in both research and disease management due to their ability to collect vast amounts of lifestyle data with high granularity and continuity [19]. While such devices provide a lower barrier to entry, some challenges regarding commercial wearable device use were reported in the studies. For example, one study in our scoping review reported that the short battery lives of smartwatches may have underestimated physical activity levels [21], and another shortlisted study reported that the Apple Watch could only collect a limited range of heart rate data [39]. Moreover, these devices are associated with data privacy concerns [39]. The “black box” algorithms typically used by most of these devices do not provide clarity on their data collection and analysis practices, leading to inherent biases and subsequent ethical drawbacks when collecting passive data [68].

Although less commonly used in the included studies, smartphone apps are useful in ecological momentary assessments through user-reported, real-time active data. This can help in self-monitoring of behaviors, symptoms, and treatment compliance, as well as in providing information/education and feedback [31]. In their review, Coghlan and D’Alfonso [13] describe a third type of data for digital phenotyping, called interactive data. These can be content-free interactions (such as swiping, tapping, and web searching) or content-rich interactions (such as social media use) [9]. For example, one of the shortlisted studies used interactive data, such as articles read per week, group posts per week, and likes per week, on an app to identify digital behavioral phenotypes of patients with obesity [59]. Such data from a smartphone can provide valuable insights into a user’s health status and behaviors, but they are also prone to data privacy concerns and inherent biases.

The use and adoption of newer analytical and machine learning methods for longitudinal data typically collected using wearables are gaining traction in digital health. We found 2 (4.3%) studies using latent class analysis [18,38], which is a statistical procedure used to identify qualitatively different subgroups within populations that share certain outward characteristics. Random forest was most common machine learning technique used [19,39,51,60], followed by logistic regression [17,51,60] and support vector machines [17,19,60]. Random forests work by combining many small, weak decisions for a single strong prediction [6]. This machine learning approach is gaining traction in noncomputational fields and is becoming a standard classification approach in many scientific fields [69]. Random forest algorithms are robust to overfitting, can deal with highly nonlinear data, and remain stable when outliers are present [70]. As 1 of our shortlisted studies reported, although neural network–based approaches outperform in unstructured data such as image and language, tree-based ensemble machine learning models such as random forests have the best performance in structured data that are essentially in tabular form [19]. One study included in our scoping review used and compared a variety of machine learning approaches, including support vector machines, k-nearest neighbors, decision trees, naive Bayes, random forest, and logistic regression; in most cases, the authors found that the random forest method worked the best [60].

Using novel machine learning approaches, passive and active data collected from wearable devices and mobile phones can be used to build “digital phenotypes,” enabling the personalization of digital health interventions and treatment plans. These digital phenotypes can be likened to customer segmentation models used by other industries. Better segmentation of health consumer behaviors can play a critical role in our ability to deliver precision digital health interventions. Some studies included in this scoping review established digital phenotypes using the digital data they collected, but these categories were not explicitly called digital phenotypes. For example, 1 study used FitBit data to classify participants into the following physical activity groups: stable active (ie, meeting physical activity recommendations for 2 weeks), stable insufficiently active, stable nonvalid wear, favorable transition (ie, improvements in the physical activity category), and unfavorable transition [33]. Another study used clinical/biometric data from a wearable sensor to develop a cough monitoring system that employed machine learning to distinguish cough and noncough units [57]. Such digital phenotypes can help “close the loop” between monitoring and taking action, helping create adaptive, tailored preventive and treatment journeys [71].

Regular use of wearable technology or behavior-tracking digital health technologies is a valuable intervention in managing health; however, personalized solutions are crucial to users' engagement, as shown by research on the use of wearables in health care [72]. Myneni and colleagues [73] analyzed the behavior change content of a community-based wearable that supports smoking cessation and found evidence from various behavior change theories, including the self-efficacy theory. Other studies examining behavior change technologies that addressed the role of self-efficacy in changing one’s behavior proposed the theory of self-efficacy as a key foundation for wearables, suggesting that perceived self-efficacy facilitates the link between intervention and behavior change [72]. Thus, integrating digital phenotyping and wearable device use can improve self-efficacy behaviors, enabling patients and health consumers to take ownership of their health and wellness.

### Future Implications

Digital phenotyping shows promise in improving person-centered care. Such precision care can help drive a proactive, predictive approach to health interventions and improved outcomes. Our scoping review highlights the increasing application of statistical and machine learning models on health consumer data from wearable devices. The opportunity to refine digital phenotypes with personal, self-reported data points and real-world passive health information is likely to add value to multiple medical research disciplines and accelerate behavioral health. The success of digital phenotyping is dependent on the willingness of hospitals, physicians, and health care organizations to participate in its development for the benefit of patients and health consumers. Hence, prospective, longitudinal studies that include larger data sets from diverse populations will be important to instill greater confidence in digital phenotyping approaches. Digital phenotyping research has been primarily explored in clinical indications related to mental illnesses and psychiatric disorders. Future work should focus on multivariate, replicable models that link to health outcomes across various indications as well as combine and analyze multiple data sources to provide a more holistic picture of an individual’s behaviors and disease state.

Furthermore, given the rapid evolution of privacy concerns affecting consumer technologies, finding ways to ensure data privacy and ethical use of health information should be seen as a strategic priority not only to understand the boundaries of the type of information that can be used for digital phenotyping but to prioritize systems and checks for health consumer consent and participation. AI and machine learning approaches need to use more transparent, replicable, bias-free algorithms to aid in robust decision making. This is especially important in low- and middle-income contexts, where legal and regulatory frameworks around machine learning deployment in health care may be inadequately defined [74].

Building digital phenotypes has tremendous opportunities in improving the user experience of mobile app–based digital health solutions, helping drive positive health outcomes. Interactive data from a smartphone can be used to generate “engagement phenotypes,” and digital journeys can be tailored to each phenotype [71]. Our previous work in machine learning suggests that metrics such as user churn combined with digital phenotyping can help improve user engagement with digital health interventions, thereby potentially leading to better outcomes [75]. Further work needs to be done on the real-world application of machine learning–based models for digital phenotyping in health care settings.

### Scoping Review Limitations

Our scoping review may have missed relevant articles because we only used 2 evidence sources (Google Scholar and PubMed) to find articles due to their open-source nature. Because we wanted to capture the breadth of digital phenotyping literature published more recently, we only considered articles published from 2020 onward. However, evidence on digital phenotyping has rapidly grown in the past couple of years. Hence, our scoping review most likely provided an apt snapshot of emerging research on digital phenotyping. For speed, multiple reviewers were involved in screening the full-text articles, which may have led to different interpretations of the results and implications. To help counteract this, we organized frequent discussions among the reviewers to address any concerns about whether a study should be included and reach a consensus. We did not conduct an in-depth citation search of the final articles. Thus, we may have missed relevant articles. Finally, we did not evaluate the quality of the included articles using validated quality assessment checklists. This was mainly due to the heterogeneity of the study characteristics.

### Conclusions

Our scoping review provides insightful foundational and application-oriented approaches toward digital phenotyping, including the use of active and passive data, differences in study design, and perhaps most importantly, the growing use of newer data analytics and machine learning algorithms to define and implement digital phenotypes in health care. Future work should focus on conducting longitudinal studies with diverse populations and larger data sets from multiple sources, leveraging newer machine learning approaches for digital phenotyping, addressing privacy and ethical concerns around passive data collection from commercial wearable devices and smartphones, and building digital phenotypes to tailor treatment plans and digital health interventions.

## Appendix

Multimedia Appendix 1 PRISMA-ScR (Preferred Reporting Items for Systematic Reviews and Meta-analyses Extension for Scoping Reviews) checklist.

Multimedia Appendix 2 Results of the data extraction and charting process of the final studies included in the scoping review.

## Abbreviations

AI: artificial intelligence
PRISMA-ScR: Preferred Reporting Items for Systematic Reviews and Meta-analyses Extension for Scoping Reviews
RCT: randomized controlled trial
